# Hair Metabolomics in Animal Studies and Clinical Settings

**DOI:** 10.3390/molecules24122195

**Published:** 2019-06-12

**Authors:** Won-Jun Jang, Jae Yoon Choi, Byoungduck Park, Ji Hae Seo, Young Ho Seo, Sangkil Lee, Chul-Ho Jeong, Sooyeun Lee

**Affiliations:** College of Pharmacy, Keimyung University, 1095 Dalgubeoldaero, Dalseo-gu, Daegu 42601, Korea; mrdoin76@gmail.com (W.-J.J.); jyoon89@hanmail.net (J.Y.C.); bdpark@kmu.ac.kr (B.P.); seojh@kmu.ac.kr (J.H.S.); seoyho@kmu.ac.kr (Y.H.S.); skdavid@kmu.ac.kr (S.L.); chjeong75@kmu.ac.kr (C.-H.J.)

**Keywords:** hair, metabolomics, chronic disease, drug addiction, mass spectrometry

## Abstract

Metabolomics is a powerful tool used to understand comprehensive changes in the metabolic response and to study the phenotype of an organism by instrumental analysis. It most commonly involves mass spectrometry followed by data mining and metabolite assignment. For the last few decades, hair has been used as a valuable analytical sample to investigate retrospective xenobiotic exposure as it provides a wider window of detection than other biological samples such as saliva, plasma, and urine. Hair contains functional metabolomes such as amino acids and lipids. Moreover, segmental analysis of hair based on its growth rate can provide information on metabolic changes over time. Therefore, it has great potential as a metabolomics sample to monitor chronic diseases, including drug addiction or abnormal conditions. In the current review, the latest applications of hair metabolomics in animal studies and clinical settings are highlighted. For this purpose, we review and discuss the characteristics of hair as a metabolomics sample, the analytical techniques employed in hair metabolomics and the consequence of hair metabolome alterations in recent studies. Through this, the value of hair as an alternative biological sample in metabolomics is highlighted.

## 1. Introduction 

**Figure 1:**
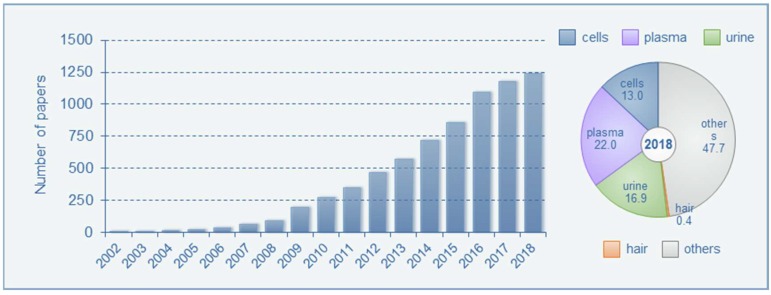
Number of Pubmed searches with the keywords, metabolomics, and biomarker, from 2002 to 2018 and the proportional contribution of biological samples in metabolomics research in 2018.

Metabolome is a term that refers to a collection of metabolites present in cells, tissues, organs, and organisms. The metabolome can be divided into three categories: All endogenous metabolites in living organisms, microbial metabolites produced by microorganisms, and all foreign metabolites derived from xenobiotics [1]. Metabolomics, a holistic analytical approach to studying metabolomes, is a powerful tool in understanding the comprehensive changes in metabolic responses in living systems induced by external stimuli or genetic alterations and is the endpoint of the omics cascade [2,3,4,5]. Unlike genomics, transcriptomics, and proteomics, metabolomics reflects the phenotype of living things, enabling us to observe simultaneous changes in many metabolites, thus aiding the discovery of biomarkers for disease diagnosis and facilitating the determination of the metabolic effects on toxicity and the exploration of the action mechanisms in pathogenesis [6,7,8,9,10,11,12,13]. The number of papers published concerning metabolomics and biomarkers has increased exponentially over the past several years. Metabolomic analysis has been performed on a variety of biological samples, including cells, plasma, urine, and tissues, with plasma (22.0%) and urine (16.9%) being the most commonly used samples in studies published in 2018 (Figure 1). Research themes were mostly related to the investigation of drug- or toxicant-induced damage and to find potential biomarkers for various diseases, such as cancer and diabetes [14,15,16,17].

Hair analysis was initially proposed as a biomonitor for chronic toxicological exposure to metals, drugs, and other toxicants. Hair is a distinct bioanalytical sample that can provide information on the history and severity of an individual’s xenobiotic exposure, based on quantitative and segmental analysis, despite the lack of pharmaco- or toxicokinetic evidence for xenobiotic deposition in hair. Substances in blood are incorporated into hair through the hair follicle and are distributed in the strands of hair as they grow [18,19,20]. In a previous study, it was proposed that xenobiotics in capillaries connected to hair roots are incorporated during melanosome transfer from melanocytes to keratinocytes as well as directly through melanocytes or keratinocytes in hair [21]. Hair pigmentation is known to be a facilitating factor for the incorporation of basic compounds. Hair, as a biomonitor, is advantageous because of its longer detection window compared to those of other biological specimens. In addition, hair has many advantages as a bioanalytical sample, including effortless sample collection, convenient transport and storage, and easily repeated sampling [18,19,20,22].

In the current review, the latest applications of hair metabolomics in animal studies and clinical settings are highlighted. For this purpose, we review and discuss the characteristics of hair as a metabolomics sample, the analytical techniques used, and the consequences of hair metabolome alterations from previous studies. Through this, the value of hair as an alternative sample in metabolomics is highlighted.

## 2. Methods

Scientific articles in the field of metabolomics and hair analysis research during the last years since 2000 were retrieved using PubMed and studied. More than twenty thousand articles were found in each research field. The search terms, ‘metabolomics’ and ‘biomarkers’ (published between 2002–2018), as well as ‘metabolomics’ and ‘cells, plasma, urine or hair’ (published in 2018), were used (Figure 1). Since our current study focuses on hair metabolomics, a PubMed search of ‘hair’ and ‘metabolomics or metabolome’ was run, and it yielded 50 articles published between 2008 and 2019. Abstracts were further examined manually in order to determine their relevance to the current review. Finally, some articles were summarized for their relevance to hair metabolomics, four for animal studies (Table 1), and eleven for clinical settings (Table 2). Additionally, relevant articles from the reference lists were considered in the discussion of hair analysis and metabolomics methodologies.

## 3. Hair as A Metabolomics Sample

### 3.1. Hair as An Analytical Sample

Human hair is composed of fibrous proteins (mostly α-keratins, 85-93%), melanins, water (typically 3–5%, and up to 15% by mass), lipids (1–9%), and mineral compounds (0.25–0.95%). Hair or fur colors are different among humans and animals depending on the composition of the melanin hair pigments which are derived from the oxidation and polymerization of tyrosine. Eumelanins and pheomelanins are the black-brown subgroup and the yellow-to-reddish brown subgroup of melanin pigments, respectively [23,24,25]. It has been reported that twenty-one proteinogenic amino acids are distributed in hair and their presence is affected by genetics, diet, hair treatments, and environmental conditions. Hair lipids originate from sebum and are composed of free fatty acids and neutral lipids [19]. Thus, hair contains functional metabolomes, such as amino acids and lipids that originate from the living body, and therefore, the metabolic changes observed in hair can be used as long-term bio-monitors for diseases or abnormal conditions. Although metabolomics studies using hair are currently limited, hair is a promising alternative sample in metabolomics. 

While there is no universal method for hair sample collection for metabolomics, the guidelines for hair sample collection for drug testing were previously published. It is recommended that a “lock of hair” or a pencil thickness of hair from at least three parts of the posterior vertex region of the scalp, where inter-individual variations such as hair growth rate and growth stage (anagen) are not significant, be collected, wrapped with a piece of aluminum foil followed by a paper envelope, and stored in a dry, dark environment at room temperature before analysis [20,26]. In previous studies on hair metabolomics, a hair sample was collected from the occipital region of the scalp [27,28,29,30,31,32,33,34,35], in the same way as hair is collected for drug testing. The collected hair was stored at either 4 °C [34] or ×20 °C [28,33,35] before analysis in metabolomics studies. 

Not only scalp hair but also hair from other parts of the body such as axillary or pubic hair were used as analytical samples, mainly in cases where scalp hair was not available or for the purpose of confirmation of the results from scalp hair analysis to prove previous drug use. However, there were inconsistencies between quantitative results from scalp hair and from hair from other parts of the body [18,36,37,38,39]. The hairs of different growth rates and different stages of hair growth (anagen, catagen, and telogen) depend on the anatomical location of hair. Sweat or sebum secretion, as a mechanism of drug incorporation, increases in axillary or pubic hair, compared with scalp hair. Moreover, there is a higher possibility of contamination depending on individual hygiene habits and lower elimination due to exposure to other external environmental conditions such as light, weather or cosmetic treatments in axillary or pubic hair [40]. 

Contamination, as well as natural or intentional variations and deterioration of both exogenous and endogenous analytes in hair, can affect the interpretation of analytical results in hair. These variable factors include personal or ethnical differences in hair pigmentation (melanin content), the external contamination by xenobiotics, and the potential washout effects from shampooing and other treatments. [18,19]. These could possibly cause pharmaco- or toxico-kinetic changes in hair. Nevertheless, previous studies have reported the overall positive correlation between drug doses and hair concentrations [41,42,43]. Moreover, statistical evaluations of the concentration of the drug and metabolites in hair from large populations have resulted in reference ranges for the severities of drug abuse [38,44]. These results suggest the potential of hair analysis in metabolomics.

### 3.2. Application of Hair Analysis in Metabolomics

As shown in Figure 1, metabolomics studies have been mostly conducted using biofluids, such as plasma or urine, from animals and humans. Hair metabolomics studies represent only 0.4% of the total papers published in 2018, which is the highest recorded in the last decade. While hair analysis has been used to investigate xenobiotic exposure over the last few decades, the analysis of the endogenous compounds, cortisol, and cortisone, in human hair was first performed by Raul et al. [45] in 2004 to monitor the use of glucocorticoids by athletes. Since then the applications of hair cortisol analysis have notably expanded and it is now used as a bio-monitor for Cushing syndrome, adrenal insufficiency, therapy monitoring, cardiovascular disease, stress, mental illness, and childhood obesity [46,47,48]. However, there are some limitations to using hair as a bio-monitor due to inter-individual variables, including hair pigmentation, hair treatment, and external contamination, which makes it difficult to provide significant correlations between the levels of hair metabolomes and disease or disorder severity [19,21,49]. Nevertheless, the clinical uses of hair cortisol concentrations demonstrate the potential of metabolomic analysis of hair and its application as a diagnostic tool for diseases or abnormal conditions.

Segmental analysis of scalp hair provides information on the history of drug or toxicant exposure or toxicological changes over time [19,50,51]. The knowledge that scalp hair grows approximately 1 cm/month allows one to predict the period during which the drug was ingested, which has been often applied to test hair samples from drug-facilitated crimes [52,53,54,55] and post-mortem drug poisoning cases [36,38,56,57] for forensic purposes. However, the determination of drug exposure time is not straightforward due to axial diffusion during segmental hair analysis [58]. Therefore, as a practical way of determining drug exposure time, analyzing each ~1 cm hair segment and comparing the results across samples was recommended. In particular, this was suggested for drugs that were also present endogenously in hair (e.g., γ-hydroxybutyrate) [59,60]. In a previous study, the long-term steroid profiling of cortisol, cortisone, testosterone, androstenedione, dihydroepiandrosterone sulphate, and 17-α-hydroprogesterone was performed in proximal 3 cm hair segments, corresponding to the history of the most recent three months. Furthermore, 3–6 cm and 6–9 cm segments, each corresponding to an additional two trimesters, were analyzed to assess the variety of steroid concentrations along consecutive segments. This study provided a potential application of hair segmental analysis in clinical endocrinology [61]. In a recent metabolomics study, the physiological transition from early to late pregnancy was successfully monitored based on the results obtained from the analysis of maternal hair segments, corresponding to each of the three trimesters [33]. Thus, segmental hair analysis can be useful for tracking changes in metabolism over time.

## 4. Analytical Techniques for Hair Metabolomics

### 4.1. General Metabolomics Methodology

In general, there are three analytical approaches in metabolomics: metabolic profiling, metabolic fingerprinting, and metabolic footprinting. Metabolic profiling is a targeted approach that involves the analysis of metabolites with similar physico-chemical properties (e.g., carbohydrates, amino acids, organic acids, nucleosides) or within the same biochemical pathways (e.g., glycolysis, gluconeogenesis, β-oxidation, citric acid cycle). Metabolic fingerprinting is an untargeted approach that investigates overall changes of metabolites in cells, tissues, and organisms. Metabolic footprinting is a comprehensive analytical approach focusing on metabolites that are secreted by cells into a specific medium rather than within the intracellular metabolome [9]. 

Several analytical tools, such as nuclear magnetic resonance spectroscopy (NMR), liquid chromatography mass spectrometry (LC-MS), gas chromatography mass spectrometry (GC-MS), capillary electrophoresis-mass spectrometry, and direct infusion mass spectrometry, are available for metabolomic studies. NMR has the advantage of reproducibility, but has lower sensitivity than that of GC-MS or LC-MS. Conversely, GC-MS has a relatively high sensitivity, but covers a limited number of metabolites with a lower range of polarity. LC-MS is considered a powerful tool for metabolomics, as it is sensitive and covers analytes with a wide range of polarity. Moreover, it can rapidly quantify a number of metabolites following a relatively simple sample preparation (i.e., no chemical derivatization required) [62,63]. The application of NMR or GC/MS in metabolomics has also been expanded with their advantages as analytical tools [2,64,65,66,67,68,69]. Elaborate sample preparation is not needed for NMR analysis. In particular, the analysis of compounds that are difficult to ionize in MS is straightforward. NMR enables compounds with identical masses, including those with different isotopomer distributions, to be identified. Recently, high sensitivity is achieved with the development of ultra-high-field NMR [70]. GC-MS has great advantages for volatile organic compounds [67]. Moreover, well-established libraries of both commercial and in- house metabolite databases are available, and the quality of matching is fairly high [71]. The use of NMR, GC/MS, and LC/MS in combination could produce complementary data and synergically investigate metabolic changes.

**Figure 2:**
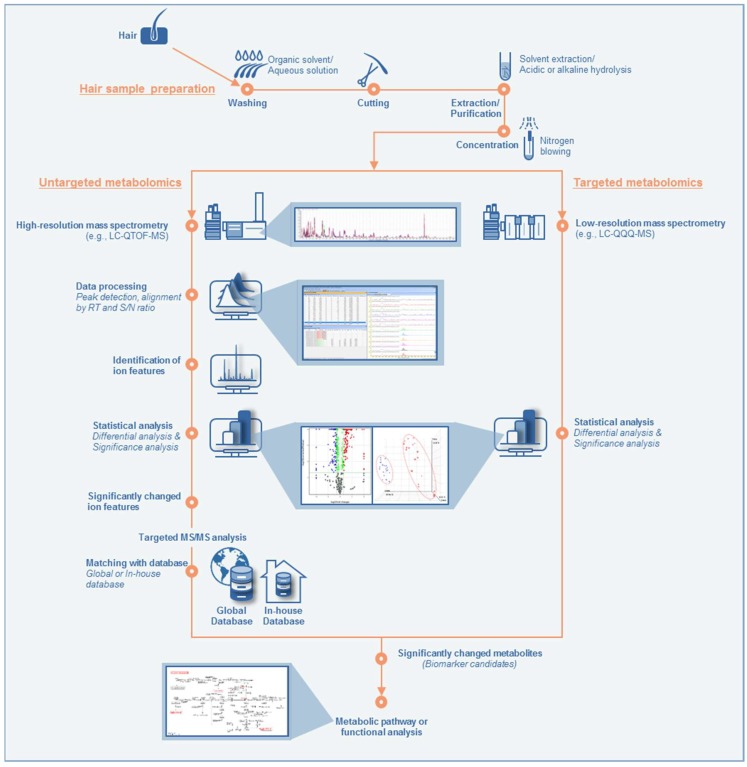
Workflow of untargeted and targeted approaches in hair metabolomics. LC-QTOF-MS, liquid chromatography-hybrid quadrupole time-of-flight mass spectrometry; LC-QQQ-MS, liquid chromatography-triple quadrupole mass spectrometry; RT, retention time; S/N ratio, signal-to-noise ratio.

For MS-based metabolomics, two different approaches are often used, namely untargeted and targeted metabolomics (Figure 2), both of which have advantages and disadvantages. In untargeted metabolomics, where a high-resolution mass spectrometer is commonly used, full-scan MS analysis followed by MS/MS analysis for the selected ion features are performed in order to identify significantly changed ion features and assign metabolites. First, the overall patterns of metabolic disturbances are investigated, based on large amounts of information from full-scan MS spectra, using differential analysis among groups of biological samples under different conditions. Metabolite assignment is commonly done by matching with public MS and/or MS/MS databases such as Human Metabolome Database (HMDB) and METLIN as well as in-house ones if available. Significantly up- or down-regulated metabolites (or ion features) and their related metabolic pathways can then be determined by significance analysis followed by pathway analysis [72,73]. This approach is mostly used for mechanistic studies, hypothesis generation, biomarker discovery, and diagnostics. However, the main problem with this approach is that it is a complicated and time-consuming process, because it deals with large amounts of raw data, and has difficulties in identifying unknown small molecules [62]. In particular, the acquisition of informative MS/MS spectra of ion features is limited due to the soft ionization of atmospheric pressure ionization and multi-adduct formation occurring in LC-MS. Moreover, MS/MS fragmentation patterns are generally inconsistent due to varying instrument conditions, limiting the applications of MS/MS spectral libraries in LC-MS. Furthermore, MS/MS spectral libraries are restricted mostly to typical metabolites and drug or toxicant-derived metabolites, making it difficult to recognize meaningful metabolites [62,74,75]. 

In contrast, in targeted metabolomics, where a low-resolution mass spectrometer is mostly employed, specific numbers of metabolites are analyzed and quantified absolutely or relatively. Therefore, to use this approach, information such as the chemical structure and molecular weight of the metabolites to be analyzed should be investigated in advance [62]. This approach is useful in understanding specific metabolic enzymes and alterations in kinetics, end products, and the known biochemical pathways of the resulting metabolic changes. When using targeted metabolomics, sample preparation can be optimized to reduce significant analytical interferences [73]. This approach has a high degree of accuracy and precision as targeted metabolites are measured with well-validated methods [72]. However, the biggest limitation of this method is that it cannot be used to identify new biomarkers, as it can only quantify previously known metabolites [62]. 

### 4.2. Sample Preparation and Instrumental Methods for Hair Metabolomics

GC-MS and LC-MS are popular analytical tools for hair analysis. For the preparation of hair samples for GC-MS or LC-MS analysis of xenobiotics or exogenous and endogenous metabolites, no specific reference method is available. In general, the hair sample preparation process consists of washing, cutting, extraction, purification, and/or concentration. The washing step is required to remove foreign matter deposited on the hair strands from the external environment. Both organic solvents (e.g., dichloromethane, acetone, methanol) and aqueous solutions (e.g., 0.1% sodium dodecylsulfate in water, distilled water) are used either alone or in sequences containing different solvents and/or aqueous solutions for decontamination. After that, analytes are extracted from the hair using a variety of methods, including incubation in organic solvents, such as methanol, and acidic or alkaline hydrolysis, depending on the chemical properties of the analytes [18,20].

Unlike other traditional biological specimens, such as blood and urine, hair is a complicated solid matrix in which compounds are firmly incorporated. Therefore, recovery or extraction efficacy is an important analytical issue in hair analysis. The extraction efficacy of a drug and a metabolite (e.g., methamphetamine and amphetamine) in hair was examined using hair reference materials, which are essential in the development of hair drug analysis methods [76,77]. However, hair reference material for endogenous compounds and standardized analytical methods in hair metabolomics are currently not available. In the previous hair metabolomics studies (shown in Table 1 and Table 2), the commonly used sample preparation methods such as LC-MS and GC-MS, i.e., general extraction, purification and/or chemical derivatization [18,20] were used for targeted or untargeted metabolomics, in the analysis of a variety of drugs and their metabolites in hair. For targeted metabolomics of polyamine, steroids, amino acids, and fatty acids, chemical derivatization for a functional group in the targeted compounds were employed [27,78,79]. One previous study conducted hair extraction using CHCl_3_ followed by evaporation, and reconstitution with CDCl_3_ for NMR-based hair metabolomics [80]. 

For LC-MS-based hair metabolomics, solvent extraction is often performed with or without an ultrasonic bath, e.g., ultrasonication using methanol and 5 M HCl (20:1) for one hour [81,82], 2 mM ammonium formate and methanol (50:50) for two hours [30] or methanol incubation for 16 hours [83]. Chemical derivatization is used specifically for the analysis of polar metabolites (e.g., amino acids) and to convert them to less polar analytes, making them more suitable for the non-polar LC stationary phase. Acidic (using 6 M HCl for all amino acids, except for tryptophan) or alkaline (using 4 M NaOH for tryptophan) hydrolysis was performed for the extraction of amino acids from human hair, and then amino acid derivation was conducted using Waters AccQ•Tag reagents [79]. For extraction of hair lipids, a previously reported systemic method based on the characteristics of lipids was employed. Solvent extraction using chloroform–methanol 2:1, 1:1 and 1:2 (v/v) was used for extractable lipids from hair and the delipidized hair was further saponified and extracted using chloroform to extract integral lipids [84]. For the identification of fatty acids in hair, chemical derivatization was performed using the 2-picolylamine, which reacts with the carboxylic acid group in fatty acids [85].

Chemical derivatization was more often employed after extraction in order to improve the volatility and sensitivity of the analytes in GC-MS-based hair metabolomics. Hair polyamines were determined to be N-ethoxycarbonyl (EOC)-N-pentafluoropropionyl (PFP) derivatives, based on the extractive two-phase EOC reaction of amino groups in aqueous solutions combined with subsequent PFP derivatization of the remaining active hydrogen atoms [78]. Hair steroids were determined after ultrasonication in methanol followed by trimethylsilylation using N-methyl-N-trifluorotrimethylsilyl acetamide, ammonium iodide, and dithioerythritol [27,31]. Also, hair samples were often derivatized using methyl chloroformate (MCF) for GC-MS-based metabolomics. MCF converts amino and non-amino organic acids (e.g., fatty acids) into volatile carbamates and esters [28,29,32,33,34,35].

## 5. Use in Animal Studies

Well-controlled animal models offer inherent phenotypes for specific diseases or abnormal conditions. Therefore, biological samples from animal models are often used in metabolomics studies [86,87,88]. However, only a few metabolomics studies have been conducted using animal hair. Table 1 summarizes the metabolomics studies performed to investigate metabolic signatures and to discover biomarkers in fur from animal models of chronic diseases, such as cardiovascular disease, diabetes, and drug addiction [81,82,83]. In a previous study using ultra-performance liquid chromatography with electrospray ionization time-of-flight mass spectrometry (UPLC-ESI-TOF-MS), the ionic features detected in fur from spontaneously hypertensive rats (SHR/Izm) and stroke-prone SHR rats (SHRSP/Izm) were compared with those of normal Wistar Kyoto control rats with advancing ages from 5 to 43 weeks. The most significantly altered ionic feature was the m/z 235.40 ion at 2.30 min, which was suggested as a potential biomarker for stroke [81]. In another previous study, the concentrations of 6 and 15 metabolites or ion features significantly increased and decreased, respectively, with age, in diabetic mice fur. N-acetyl-L-leucine detected in fur, together with other biological samples, such as plasma, liver, and kidney, from diabetic mice, was suggested as a potential biomarker based on metabolomic profiling results [82]. Choi et al. conducted metabolic characterizations in urine and fur from a rat model of methamphetamine self-administration. In the rat fur samples, some functional metabolites, including acetylcarnitine, palmitoyl-(l)-carnitine, deoxycorticosterone, oleamide, and stearamide, significantly changed, which implies metabolic perturbations in the central nervous system and energy production. Since the more significantly changed functional metabolites were observed in fur compared with urine, animal hair was proposed as a more reliable diagnostic specimen for drug addiction [83]. Moreover, drug-induced sebaceous gland atrophy was examined using fur from rats and hamsters. Since animal hair is coated with sebum and reflects dermal condition, animal hair metabolomics was suggested as a test method for dermal toxicity [80].

## 6. Use in Clinical Settings

Previous hair metabolomic investigations in clinical settings are summarized in Table 2. Mostly, clinical hair metabolomics was applied for the long-term monitoring of chronic pathophysiological conditions (e.g., pregnancy complications, cancer, male pattern baldness, drug addiction, etc.). Those previous studies demonstrated that human hair is a potential diagnostic sample that contains robust and stable biomarkers for chronic diseases [27,28,29,30,31,32,33,78].

The clinical applications of maternal hair metabolomics recently increased, in particular to discover diagnostic biomarkers and study metabolic mechanisms in pregnancy-related complications, such as fetal growth restriction (FGR), small-for-gestational-age infants (SGA), gestational diabetes mellitus (GDM), infant lower language ability, and intrahepatic cholestasis of pregnancy (ICP) [28,29,32,33,34,35]. Some key hair metabolites, up- or down-regulated with pregnancy-related complications, are listed with their implications in Table 2. A loss of redox control and a deficiency of precursors for fetal development and growth were linked to FGR [28]. Previous studies on the changes of GDM-related hair metabolomes reported lipid peroxidation related to the oxidative stress environment in diabetes, deficits in energy metabolism, and degradation of amino acids, but no correlation between maternal diet and hair metabolomes [29,33,34]. Deplancke et al. also found that the concentrations of margaric acid, pentadecanoic acid, and myristic acid in hair from pregnant women with SGA significantly decreased, which implies a deficit in placental function of fatty acid transfer to the fetus [33]. Recently, de Seymour et al. examined the hair metabolomics of ICP, with no correlation between ICP and hair metabolomes observed [35]. Furthermore, another maternal hair metabolomics study demonstrated the correlation between infant lower language ability and higher maternal phthalate exposure [32]. 

The metabolic profiling for classes of targeted analytes (e.g., polyamines, steroids, sterols) in human hair was employed in patients with cancer [78], male pattern baldness [27], and cognitive impairment or Alzheimer’s disease [31]. Those studies clarified the relationship between disease or abnormal conditions and known metabolic pathways, and provided information on the metabolic basis of those diseases. In hair from patients with cervical cancer or ovarian cancer, significant increases in the levels of putrescine, spermidine, and spermine were observed, probably due to deficits in polyamine biosynthesis and accumulation in hair [78]. The metabolic alteration in male-pattern baldness, and the metabolic effects of dutasteride, an inhibitor of 5α-reductase, were successfully investigated by hair steroid profiling [27]. Son et al. proved that cognitive impairment was caused by the up-regulation of 7α- and 7β-hydroxycholesterol due to impaired cholesterol metabolism and suggested the latter as a predictive biomarker [31].

Hair damage by dyeing, perming, and bleaching was evaluated by absolute quantification of the amino acids and lipids in hair. The hair concentration of cysteic acid and cysteine/cysteic acid increased while methionine and tryptophan decreased significantly with hair treatment. Hair lipids, such as erucic acid, behenic acid, lignoceric acid, nervonic acid, cerotic acid, and 18-methyl eicosanoic acid, were also down-regulated [79]. 

Drug addiction is a chronic relapsing disorder which develops from the repetition of positive and negative effects caused by temporary drug use and withdrawal, respectively [89,90]. Clinical laboratory criteria for the diagnosis and treatment of drug addiction are not fully elucidated, and no specific biomarkers are currently available. Only limited information is available regarding the effect of drug abuse or addiction on endogenous metabolites in hair. Xie et al. previously reported that sorbitol and cortisol were up-regulated while arachidonic acid, glutathione, linoleic acid, and myristic acid were down-regulated, based on a metabonomic study on heroin addicts hair. This result implies that energy metabolism, sorbitol pathway, and immune cell function are disturbed by heroin addiction [30].

## 7. Conclusions

**Figure 3:**
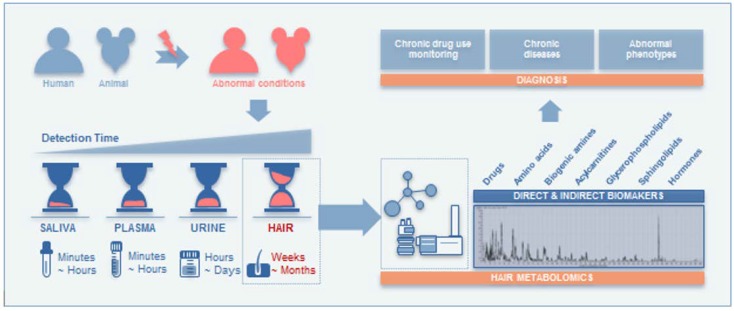
Application of hair metabolomics.

As shown in Figure 3, hair metabolomics approaches have recently been employed to investigate the metabolic signature of a variety of chronic diseases or abnormal conditions in animal studies and clinical settings to explain the pathophysiological mechanisms underlying disease, as well as to propose new diagnostic biomarkers for long-term monitoring. Hair has a great advantage in that the endogenous compounds deposited in hair are retained. Metabolic alterations identified in hair could provide insight into metabolic perturbation over a longer period of time than other conventional biological samples (e.g., plasma, urine). Thus, hair metabolomics can be a successful metabolomics approach with high potency for evaluating the animal and human pathological conditions.

## Figures and Tables

**Table 1:** Use of hair metabolomics in animal studies.

No.	Pathologic Condition	Study Objective	Study Subjects (Animal Species, Animal Model, etc.)	Sample Preparation	Analytical Platform (Untargeted or Targeted)	Key metabolites Changed (Possible Biomarkers)		Consequence of Metabolic Changes	Reference (Year Published)
						Increased	Decreased		
1	Stroke	Biomarker discovery	Spontaneously hypertensive rats (SHR/Izm) and stroke-prone SHR rats (SHRSP/Izm)	Acidic solvent sonication	UPLC-ESI-TOF-MS (Untargeted)	m/z 235.40 ion at 2.30 min	-	Potential biomarker of stroke	Inagaki et al., J Chromatogr A (2007)
2	Diabetes	Biomarker discovery	Spontaneous insulin-resistant mice (ddY-H)	Brief solvent extraction	UPLC-ESI-TOF-MS (Untargeted)	-	N-Acetyl-L-leucine	Potential biomarker of diabetes	Tsutsui et al., Clin Chim Acta (2011)
3	Dermal toxicity (drug-induced sebaceous gland atrophy)	Metabolic profiling	Rats and hamsters dosed with a stearoyl-CoA desaturase 1 (SCD 1) inhibitor	Solvent incubation for 16 h	NMR (Untargeted)	-	1,2-distearoyl-3-oleoyl-rac-glycerol, lathosterol-like sterol esters, wax ester, total cholesterol, and cholesterol for rats, and lathosterol-like sterol esters, and wax ester for hamsters	Reduction of lipid levels by an SCD 1 inhibitor	Khandelwal et al., J Lipid Res (2014)
4	Drug addiction	Metabolic profiling	Methamphetamine self-administering rats	Solvent incubation	UPLC-ESI-QTOF-MS (Untargeted)	Acetylcarnitine, 5-methylcytidine, 1-methyladenosine, palmitoyl-(l)-carnitine	(l)-Norvaline/betaine/5-aminopentanoate/(l)-valine, lumichrome, deoxycorticosterone, oleamide, stearamide, hippurate	Metabolic perturbation in the central nervous system and energy production	Choi et al., Metabolomics (2017)

UPLC-ESI-TOF-MS, ultra-performance liquid chromatography with electrospray ionization time-of-flight mass spectrometry; NMR, nuclear magnetic resonance spectroscopy; UPLC-ESI-QTOF-MS, ultra-performance liquid chromatography with electrospray ionization quadrupole time-of-flight mass spectrometry.

**Table 2:** Use of hair metabolomics in clinical settings.

\	Pathologic Condition	Study Objective	Study Subject (Age, Gender, Number of Subjects, etc.)	Sample Preparation	Analytical Platform (Untargeted or Targeted)	Key Metabolites Changed (Possible Biomarkers)		Consequence of Metabolic Changes	Reference (Year Published)
						Increased	Decreased		
1	Cancer	Polyamine measurement for cancer diagnosis	Patients with cervical cancer (34–65 yr, n = 13) or ovarian cancer (37–75 yr, n = 11)	Acidic solution incubation followed by N-ethoxycarbonylation and N-pentafluoropropionylation	GC-MS (Targeted)	Putrescine, spermidine, spermine	-	Deficits in polyamine biosynthesis and accumulation	Choi et al., Clin Chem (2001)
2	Male pattern baldness	Steroid profiling	Balding men (32.5 yr (mean), n = 19)	Solvent sonication followed by solid phase extraction and trimethylsilation	GC-MS (Targeted)	Dihydrotestosterone, dihydrotestosterone/testosterone ratio, and cortisol/cortisone ratio	-	Increase of 5α-reductase activity	Jung et al., Rapid Commun Mass Spectrom (2011)
3	Fetal growth restriction	Biomarker discovery	Pregnant women (22–44 yr, 26–28 weeks of gestation, n = 41)	Alkaline hydrolysis followed by chemical derivatization with methyl chloroformate	GC-MS (Untargeted)	Heptadecane, NADPH/NADP, saturated fatty acids (palmitate, 2-methyloctadecanoate, myristate, margarate, stearate, dodecanoate, and octanoate)	Amino acids (lysine, methionine, tyrosine, valine, and threonine), glutathione	Loss of redox control and deficiency of precursors for fetal development and growth	Sulek et al., Theranostics (2014)
4	Gestational diabetes mellitus (GDM)	Biomarker discovery	Pregnant women (30 yr (median), 26–28 weeks of gestation, n = 94)	Alkaline hydrolysis followed by chemical derivatization with methyl chloroformate	GC-MS (Untargeted)	Adipic acid	-	Lipid peroxidation related to the oxidative stress environment in diabetes	He et al., Acta Diabetol (2016)
5	Hair damage by dyeing, perming, and bleaching	Metabolic profiling	Treated samples of natural hair of Asians (Beaaulax Co., Saitama, Japan, n = 10)	Acidic or alkaline hydrolysis followed by chemical derivatization with Waters AccQ•Tag reagents for amino acids	UPLC-PDA and	Cysteic acid and cysteine/cysteic acid	Methionine and tryptophan	Quantitative grading of hair damage	Joo et al., Exp Dermatol. (2016)
				Solvent extraction for extractable lipids and further saponification and solvent extraction followed by chemical derivatization with 2-picolylamine for fatty acids	UPLC-ESI-QQQ-MS (Targeted)	-	Erucic acid, behenic acid, lignoceric acid, nervonic acid, cerotic acid, and 18-methyl eicosanoic acid		
6	Heroin addiction	Metabolic profiling	Heroin abusers (20–56 yr, male, n = 40, female, n = 18)	Solvent sonication	UFLC-ESI-IT-TOF-MS (Untargeted)	Sorbitol and cortisol	Arachidonic acid, glutathione, linoleic acid, and myristic acid	Deficits in energy metabolism, sorbitol pathway, and immune cell function	Xie et al., J Mol Neurosci (2016)
7	Cognitive impairment	Sterol profiling	Patients with mild cognitive impairment (MCI, 70.3 yr (mean), female, n = 15) or Alzheimer’s disease (70.8 yr (mean), female, n = 31)	Solvent pulverization followed by trimethylsilation	GC-MS (Targeted)	7α-Hydroxycholesterol and 7β-hydroxycholesterol	-	Impaired cholesterol metabolism	Son et al., J Steroid Biochem Mol Biol (2016)
8	Infant lower language ability	Maternal hair metabolic profiling for infant neurodevelopment	Pregnant women (26–28 weeks of gestation, n = 373)	Alkaline hydrolysis followed by chemical derivatization with methyl chloroformate	GC-MS (Untargeted)	Phthalic acid	-	Infant lower language ability caused by high maternal phthalate exposure	Jones et al., Sci Rep 2018
9	Small for gestational age infants	Biomarker discovery and metabolic mechanism study	Pregnant women (30.8 yr (mean), 39.1 weeks of gestation, n = 20)	Alkaline hydrolysis followed by chemical derivatization with methyl chloroformate for GC-MS and alkaline hydrolysis followed by solvent extraction for LC-MS	GC-MS and UPLC-ESI-QTOF-MS (Untargeted)	Margaric acid, pentadecanoic acid, and myristic acid	-	Deficits in placental function of fatty acid transfer to the fetus	Delplancke et al., Sci Rep (2018)
	GDM		Pregnant women (32.7 yr (mean), 38.6 weeks of gestation, n = 11)			1-Hydroxy-3-nonanone and 22-oxavitamine D3	Tryptophan, leucine, citric acid, 3,4-oxaolidinercarboxylic acid, 2-oxovaleric acid, 3-pyridinecarboxamide, 2-methylpentan-2-yl trifluoraoacetate, and 2-oxobutyric acid	Deficits in energy metabolism and degradation of amino acids	
10	GDM	Maternal hair metabolic profiling for gestational diabetes mellitus	Pregnant women (32 yr (mean), 24–28 weeks of gestation n = 49)	Alkaline hydrolysis followed by chemical derivatization with methyl chloroformate	GC-MS and UPLC-ESI-QTOF-MS (Untargeted)	Pentachloroethane, 1-hydroxyvitamin D5, (3beta,23E)-3-hydroxy-27-norcycloart-23-en-25-one, (4-methylphenyl)acetaldehyde, linalyl isobutyrate, and 3-phenyl-1-propanol	Proline, 4-methoxy-benzoic acid, 5-methylhexanoic acid, dihydroceramide, 2,2,9,9-tetramethyl-undecan-1,10-diol, palmitoylglycine, benzeneacetic acid, 2-butenoic acid, glutamic acid, but-2-enedioic acid, 2-oxobutyric acid, N,4-diethyl-4-heptanamine, N-methoxycarbonyl-l-proline, pyrrolidine-1,2-dicarboxylic acid, (1-ethyl) ester, NADP_NADPH, malonic acid, 2-methylcyclohexanone, 3-hydroxy-2-octanone, and C17 sphinganine	No correlation between maternal diet in GDM and hair metabolomes	Chen et al., Metabolomics (2018)
11	Intrahepatic cholestasis of pregnancy (ICP)	Biomarker discovery	Pregnant women (27.9 yr (mean), 17-41 weeks of gestation n = 38)	Alkaline hydrolysis followed by chemical derivatization with methyl chloroformate	GC-MS	-	Adipic acid and succinic acid	No correlation between ICP and hair metabolomes	de Seymour et al., Metabolomics (2018)

yr, years; GC-MS, gas chromatography mass spectrometry; UPLC-PDA, ultra-performance liquid chromatography photodiode array detector; UPLC-ESI-QQQ-MS, ultra-performance liquid chromatography with electrospray ionization triple quadrupole mass spectrometry; UFLC-ESI-IT-TOF-MS, ultra-fast liquid chromatography with electrospray ionization ion-trap-time of flight mass spectrometry; UPLC-ESI-QTOF-MS, ultra-performance liquid chromatography with electrospray ionization quadrupole time-of-flight mass spectrometry
